# Linking Fruit Ca Uptake Capacity to Fruit Growth and Pedicel Anatomy, a Cross-Species Study

**DOI:** 10.3389/fpls.2018.00575

**Published:** 2018-05-09

**Authors:** Wenpei Song, Junwen Yi, Odit F. Kurniadinata, Huicong Wang, Xuming Huang

**Affiliations:** ^1^College of Horticulture, South China Agricultural University, Guangzhou, China; ^2^Department of Agronomy and Horticulture, Faculty of Agriculture, Bogor Agricultural University, Bogor, Indonesia

**Keywords:** calcium transport pathway, calcium uptake rate, calcium uptake activity, xylem functionality, vessel characters, fruit growth

## Abstract

Calcium (Ca) in flesh fruits is important for quality formation and maintenance. Most studies on fruit Ca focus on one species. This study attempted to understand some universal relations to fruit Ca uptake across species. Calcium contents in fruit tissues were analyzed in different fruits, including three cultivars of litchi, two cultivars each of grape and citrus, and one cultivar each of loquat, apple, pear, Indian jujube, and longan. *In situ* Ca distribution was revealed with electron probe and xylem functionality visualized by dye tracing. Fruit Ca uptake rate and activity were calculated and correlated with fruit growth and pedicel anatomy. The results showed that fruit Ca uptake rate was the highest in pomes (loquat, apple, and pear), followed by Indian jujube drupe, arillate fruits (litchis and longan) and citrus, while grape berries were the lowest. Fruit Ca uptake rate showed a strong positive correlation to growth rate. However, Ca uptake activity, reflecting Ca uptake rate relative to growth, was the highest in arillate fruits and loquat and lowest in grape berries, and had a poor correlation with fruit growth rate. In all fruits, Ca concentration in the pedicel was higher than in the fruit, and they displayed a good positive correlation. In the pedicel, Ca was most abundant in the phloem. Dye tracing showed that xylem function loss occurred with maturation in all species/varieties. Apple had the poorest xylem functionality with the least development of secondary xylem, but its Ca uptake rate was among the highest. Vessel density, size and area in the pedicel showed no correlation with fruit Ca uptake rate. It is concluded that: (1) fruit growth may be a key determinant of Ca uptake; (2) the universal pattern of Ca being higher in the pedicel than in the fruit indicates existence of a pedicel-fruit “bottleneck” effect in Ca transport across species; (3) xylem functionality loss with fruit maturation is also a universal event; (4) in the pedicel, Ca is more distributed in the phloem; (5) vessel morphology in the pedicel is not rate-limiting for fruit Ca uptake; (6) phloem pathway might contribute to fruit Ca uptake.

## Introduction

In plant, calcium (Ca) has irreplaceable functions such as construction of cell wall and signal transduction involving responses to external or internal signals (White and Broadley, 2003; Hocking et al., 2016). Studies have shown that systemic or localized Ca deficiency in plants causes various physiological disorders leading to substantial yield and quality losses (Shear, 1975; Huang et al., 2005a; Saure, 2005; Yang and Jie, 2005; Tonetto de Freitas and Mitcham, 2012). Fruit, a terminal organ with low transpiration, is more vulnerable to shortage of Ca supply (White and Broadley, 2003). However, development of Ca deficiency symptoms is determined by a number of internal and external factors including transport of Ca to fruit, Ca allocation and subcellular distribution (Bangerth, 1979; Ho and White, 2005; Tonetto de Freitas et al., 2014; Hocking et al., 2016).

Fruits are architecturally isolated organs connected to tree by pedicel/peduncle, where sap carrying various nutrients is fed to fruit via xylem and phloem. The transport of Ca to fruit in the pedicel does not seem smooth, as our studies showed that Ca concentration in the pedicel/peduncle was 10 times higher than in fruit in litchi, reminiscent of a “bottleneck effect” in Ca transport to the fruit (Huang et al., 2006; Song et al., 2018). If such bottleneck effect exists, then the more Ca moves into fruit, the more Ca is sequestered in the pedicel, and a positive correlation between fruit Ca uptake and Ca concentration in the pedicel will thus be expected. We are interested to examine the pedicel-fruit Ca gradient in a wider range of fruit types and analyze the correlation between fruit Ca uptake and Ca sequestration in the pedicel.

Although it is generally believed that Ca transport is exclusively through the xylem/apoplast pathway (Ferguson, 1980; Hanson, 1984; Zocchi and Mignani, 1995; Saure, 2005; Hocking et al., 2016), there are some studies showing that phloem can be an important pathway for Ca delivery (Stebbins and Dewey, 1972; Himelrick and McDuffie, 1983; Vemmos, 2005; Song et al., 2018). We recently found Ca content in the phloem was much higher than in the xylem in litchi pedicel, indicative of possible Ca deliver to fruit via phloem mass flow (Song et al., 2018). It is not known whether this Ca distribution pattern in the pedicel is universal in different fruit species.

The xylem-based Ca transport is influenced by factors including the rate of xylem sap inflow, the competition between ions for binding sites in xylem vessel walls and pit membranes, and sequestration by organic acids like oxalate (Franceschi and Nakata, 2005; Saure, 2005; Gilliham et al., 2011; Hocking et al., 2016). The xylem inflow resulting from fruit transpiration and fruit growth is a key determinant for fruit to acquire Ca (Montanaro et al., 2014, 2015; Tonetto de Freitas et al., 2014. Fruit transpiration is a function of vapor pressure deficit (VPD) (Montanaro et al., 2012). Montanaro et al. (2015) showed that fruit Ca gain in kiwifruit is coupled to transpiration at high VPD that induces high fruit transpiration, while at low VPD with low fruit transpiration, fruit growth dominates Ca uptake. Xylem inflow rate is also a function of hydraulic conductivity, which is determined by xylem functionality and positively related to vessel diameter and frequency. In fruits like apple (Miqueloto et al., 2014), grape (Coombe and McCarthy, 2000; Choat et al., 2009) and kiwi (Dichio et al., 2003), loss of xylem functionality occurs during fruit development, especially in late stages. In apple, time of xylem functionality loss is related to susceptibility to bitter pit, a disorder caused by Ca deficiency (Miqueloto et al., 2014). Xylem conductivity is also determined by xylem anatomic features in the pedicel, which theoretically have an impact on fruit Ca uptake. However, there is a lack of study exploring this impact.

Previous studies of fruit Ca focused on one species. Different cultivars of the same species, apple (Miqueloto et al., 2014) and litchi (Huang et al., 2006) for example, show great difference in fruit Ca uptake and thus in susceptibility to disorders caused by Ca deficiency. Therefore, genetic background is a key determinant of fruit Ca uptake capacity. However, there is little information about fruit attributes related to the difference in Ca uptake capacity cross species. In this study, a cross-species analysis of fruit Ca uptake capacity was carried out in relation to pedicel anatomy and xylem functionality as well as to fruit growth rate in order to generalize some universal relations to fruit Ca uptake capacity across fruit species.

## Materials and Methods

### Materials

Twelve varieties of 8 fruit species, including 3 cultivars of litchi, 2 cultivars each of grape and citrus, and 1 cultivar each of loquat, apple, pear, jujube, and longan, were chosen as the experimental materials for this study. Information of the fruits and their abbreviated codes are listed in **Table 1**. Mature fruit with stalk of each variety were harvested from at least 3 trees on the dates shown in **Table 1**. For convenience, the codes of the materials are used in the following text. Apple (Md-TM1) and pear (Pp-WK) samples were collected from the experimental orchard of the Horticultural Institute, Guizhou Academy of Agricultural Sciences (Guizhou, China) with a soil pH of 6.83 and available Ca around 680 mg kg^-1^; citrus (Cr-STJ and Cr-MSJ), loquat (Ej-ZZ6) and grape (Vv-SB and Vv-SM) samples were taken from the experimental orchard of South China Agricultural University (Guangzhou, China) with a soil pH of 5.47 and available Ca around 320 mg kg^-1^; litchis (Lc-LFN, Lc-GW, and Lc NMC) and longan (Dl-SX) were sampled from Xili Orchard (Shenzhen, China) with a soil pH of 5.1 and available Ca around 250 mg kg^-1^; Indian Jujube (Zm-DMS) fruit were from the experimental orchard of Tropical and Subtropical Crop Institute, China Academy of Tropical Horticultural Sciences (Zhanjiang, China) with a soil pH of 5.4 and available Ca around 400 mg kg^-1^. Changes VPD in different sites during fruit development are shown in **Supplementary Figures S1**, **S2**. The average VPDs throughout the period of fruit development of all the tested fruits are also shown in **Table 1**.

**Table 1:** Information of the experimental materials used in this study.

Materials	Code	Tree information	Flowering date	Sampling Date	Average VPD during fruit growth (kPa)
*Malus domestica* cv. Tengmu No.1	Md-TM1	8-year-old trees grafted on *Malus spectabilis*	May 1, 2015	July 5, 2015	0.696
*Pyrus pyrifolia.* cv. Whangkeumbae	Pp-WK	8-year-old trees grafted on *Pyrus betulifolia*	May 1, 2015	July 31, 2015	0.765
*Citrus reticulate* cv. Shatangju	Cr-STJ	8-year-old trees grafted on *Poncirus trifoliata*	April 10, 2015	December 26, 2015	0.548
*Citrus reticulate* cv. Mashuiju	Cr-MSJ	8-year-old trees grafted on *Poncirus trifoliata*	April 10, 2015	November 24, 2015	0.545
*Eriobotrya japonica* cv. Zaozhong No.6	Ej-ZZ6	16-year-old self-rooted trees	November 27, 2015	March 17, 2016	0.557
*Vitis vinifera* cv. Summer black	Vv-SB	3-year-old vines on Beta rootstock	April 1, 2015	June 27, 2015	0.390
*Vitis vinifera* cv. Shine Muscat	Vv-SM	3-year-old vines on Beta rootstock	April 1, 2015	July 10, 2015	0.451
*Litchi chinensis* cv. Lingfengnuo	Lc-LFN	4-year-old branches top-grafted on Huaizhi	April 8, 2016	June 23, 2016	0.395
*Litchi chinensis* cv. Guiwei	Lc-GW	18-year-old trees grafted on Huaizhi	April 7, 2016	June 26, 2016	0.395
*Litchi chinensis* cv Nuomici	Lc-NMC	18-year-old trees grafted on Huaizhi	April 7, 2016	June 26, 2016	0.395
*Dimocarpus longan* cv. Shixia	Dl-SX	18-year-old trees grafted on Chuliang	April 15, 2016	July 31, 2016	0.480
*Ziziphus mauritiana* cv. Damisi	Zm-DMS	13-year-old trees grafted on Yuenanzao	September 26, 2016	December 25, 2016	0.853


### Determination of Calcium Uptake Capacity of Fruit

Ten mature fruit with stalk were harvested from each of selected trees. The fruit were individually separated into stalk, skin, flesh, and seed and their fresh weights were collected before oven dried at 65°C for 72 h. After the dry weight of each part was recorded, the dried samples were ashed in a muffle furnace. The ashed samples were separately dissolved in 0.1 mol L^-1^ HCl solution, and Ca content was analyzed using a flame atomic absorption spectrophotometer (Z-2300, Hitachi, Japan). After Ca content in each fruit part was collected, total content of calcium in individual fruit was calculated by summing the Ca contents in seed, flesh and skin. This was further used to calculate fruit Ca uptake rate and fruit Ca uptake activity.

Fruit Ca uptake rate (μg d^-1^) = Total fruit Ca content/days of fruit development (from anthesis to harvest).

The fruit Ca uptake rate thus calculated refers to the average Ca accumulation per day per fruit during the whole period of fruit development.

Fruit calcium uptake activity (μg d^-1^ g^-1^DW) = Fruit uptake rate/fruit dry weight.

Fruit Ca uptake activity reflects Ca uptake rate relative to fruit biomass accumulation and thus shows the balance between fruit Ca uptake and fruit growth.

### Xylem Functionality Observation Using Dye Tracing

Five fruit with stalks of each species/variety were randomly collected from the trees at full maturity as well as at the early maturing phase, when fruits shifted from a utilization sink to a storage sink and started to accumulate assimilates. The exact sampling times (days after full bloom, DAFB) can be found in **Table 2**. Fruit samples were fed with 1% (w/v) safranin O solution via fruit stalk by submerging the freshly cut stalk end into the solution. Twenty four hour later, the fruit were cut into equal halves longitudinally from fruit top to fruit base. Distribution of the red dye reflecting functionality of xylem in fruit/pedicel was revealed and photographed.

**Table 2:** Times of sampling for vessel functionality observations.

Materials (common species name)	Early maturing phase	Ripe phase
Ej-ZZ6 (loquat)	DAFB 90	DAFB 110
Md-TM1 (apple)	DAFB 53	DAFB 65
Pp-WK (pear)	DAFB 49	DAFB 91
Cr-STJ (citrus)	DAFB180	DAFB 260
Vv-SB (grape)	DAFB 51	DAFB 88
Vv-SM (grape)	DAFB 51	DAFB 101
Dl-SX (longan)	DAFB 72	DAFB 107
Lc-GW (litchi)	DAFB 63	DAFB 80
Lc-NMC (litchi)	DAFB 63	DAFB 80
Zm-DMS (Indian Jujube)	DAFB 60	DAFB 90


### *In Situ* Ca Distribution in Pedicel Tissues

The structure of and Ca distribution in fruit pedicel were observed with a JXA -8100 electron probe microanalyzer. Sample preparation, observation and image processing were carried out according to Song et al. (2014). The pedicels were cut into 0.2 mm thick cross sections, stuck onto a cupper sample stand with electricity-conductive carbon glue, and coated with platinum in a JFC-1600 vacuum auto-coater for 90 s. During electron probe analysis, the working distance of all samples was set at 11 mm, with a probe accelerating voltage of 20 KV and an exciting current of 2 × 10^-8^ A. The panorama images of the pedicels of all fruits were collected at a magnification of 40. Under a magnification of 150–300, secondary the electron images of the samples and the map images of Ca-characteristic X-ray signal (Ca mapping image) were separately collected. Intensity of Ca-characteristic X-ray signal which reflects Ca abundance was displayed by density of bright dots. Adobe Photoshop CS6 was used to extract the bright dot map and change it into red color. The calcium mapping image was combined with the corresponding secondary image to generate a direct view of *in situ* Ca distribution in the tissues.

### Optical Microscopic Observation and Analysis of Pedicel Anatomy

Pedicels from the above mentioned samples were collected, cut into 2 mm pieces and fixed in FAA solution (formalin: acetic acid: 70% alcohol = 1: 1: 9 and 5 ml glycerinum). After dehydration with gradient concentrations of alcohol solutions from 70 to 100% and cleared with butyl alcohol, the samples were embedded in paraffin. Sections of 10 μm in thickness were cut with a rotary microtome (Leica, RM2235, Germany), and stained successively with safranin (1 g in 100 ml) and fast green (0.5 g in 100 ml of 95% ethanol) after rehydration with gradient alcohol solutions from 100% to 70%. The sections were observed and photographed under a ZEISS microscope equipped with AxioCam HRc Imager D2 (Germany). Vessel number in the cross sections of the pedicel was counted and the total cross area of the pedicel, the xylem area, and the vessel size were obtained with a Digimizer image analyzing software system. Three biological replicates in each fruit species/variety were used for this experiment.

### Statistical Analysis

Fruit weight and Calcium contents in various fruit tissues were collected from at least 30 individual fruit. The results are presented as means ± SE. Analyses of variation (ANOVA), Scheffe multiple range tests and correlation analyses were carried out using the statistical package of SPSS 21 (SPSS Inc., Chicago, IL, United States).

## Results and Analysis

### Ca Uptake Capacity in Different Fruit Species/Varieties

**Table 3** shows fruit development duration and fruit size differed greatly among varieties/species. With the two parameters, the average fruit growth rate throughout fruit growth period can be calculated by dividing fruit weight with duration of fruit development. Pp-WK, Md-TM1 and Zm-DMS had the highest growth rate, while grape berries (Vv-SB and Vv-SM) and Dl-SX had the lowest. Fruit Ca concentration had significant difference among the tested fruit species/varieties. The fruits could be arbitrarily divided into three groups based on Ca concentration. The low Ca fruits with Ca concentration lower than 1 mg g^-1^ DW included the two grape varieties(Vv-SB and Vv-SM), Lc-NMC, Zm-DMS, Md-TM1, and Pp-WK; the medium Ca fruits with Ca concentration between 1 and 2 mg g^-1^ DW included Lc-LFN, Lc-GW and Ej-ZZ6; the high Ca fruits with calcium higher than 2 mg g^-1^ DW included the two citrus varieties (Cr-STJ and Cr-MSJ) and Dl-SX. In the flesh tissue, the edible part of fruit, Ca concentration was the highest in Ej-ZZ6, followed in descending order by Cr-MSJ, Cr-STJ, Zm-DMS, Md-TM1, Pp-WK, Dl-SX, Vv-SM, and Vv-SB, and litchis had the lowest Ca concentration in the flesh. Therefore, loquat and citrus fruits are good source of Ca nutrition for human beings. There was no defined pattern in fruit and flesh Ca concentrations among fruit types (e.g., drupes, pomes, berries, and arillate fruits).

**Table 3:** Comparison of calcium contents in fruit and pedicel and fruit calcium uptake capacity among fruit species/varieties.

Fruit type	Cultivar	Duration of fruit growth (d)	Fresh weight (g)	Dry weight (g)	Growth rate (mg d^-1^)	Fruit Ca content (mg fruit^-1^)	Pedicel Ca concentration (mg g^-1^ DW)	Fruit Ca concentration (mg g^-1^ DW)	Flesh Ca concentration (mg g^-1^ DW)	Fruit Ca uptake rate (μg d^-1^)	Fruit Ca uptake activity (μg d^-1^ g^-1^)
Pome	Ej-ZZ6	110	40.78 ± 1.45 c	7.20 ± 0.34 cd	65.48 ± 3.11 d	13.43 ± 1.11 a	11.46 ± 0.63 b	1.91 ± 0.15 bc	2.66 ± 0.21 a	122.11 ± 10.12 ab	17.35 ± 1.38 bc
	Md-TM1	65	75.47 ± 6.09 b	8.83 ± 0.76 bc	135.86 ± 11.66 b	6.43 ± 1.18 c	3.94 ± 0.34 d	0.78 ± 0.15 de	0.75 ± 0.15 cde	100.41 ± 18.49 abc	12.14 ± 2.36 bcde
	Pp-WK	91	142.13 ± 6.70 a	16.83 ± 1.05 a	184.99 ± 11.59 a	11.97 ± 0.51 ab	9.16 ± 0.72 bcd	0.73 ± 0.04 de	0.65 ± 0.04 cde	131.51 ± 5.59 a	8.01 ± 0.47 de
Citrus	Cr-STJ	260	41.17 ± 3.29 c	6.22 ± 0.52 de	23.91 ± 2.02 e	14.69 ± 1.30 a	8.58 ± 0.42 bcd	2.38 ± 0.10 b	1.15 ± 0.08 bc	56.48 ± 5.01 de	9.15 ± 0.40 cde
	Cr-MSJ	228	29.42 ± 1.33 cd	5.38 ± 0.23 def	23.61 ± 1.00 e	13.92 ± 0.75 a	11.35 ± 0.55 b	2.62 ± 0.12 ab	1.59 ± 0.09 b	61.07 ± 3.30 cde	11.50 ± 0.53 bcde
Aril	Dl-SX	107	5.75 ± 0.23 e	1.74 ± 0.08 hi	16.25 ± 0.72 e	5.76 ± 0.36 c	17.66 ± 1.12 a	3.37 ± 0.18 a	0.53 ± 0.04 cde	53.79 ± 3.40 de	31.54 ± 1.68 a
	Lc-LFN	76	19.49 ± 0.69 d	3.75 ± 0.15 gh	49.36 ± 1.95 d	4.26 ± 0.31 cd	5.83 ± 0.42 bcd	1.13 ± 0.06 cde	0.17 ± 0.01 e	56.07 ± 4.02 de	14.89 ± 0.82 bcd
	Lc-GW	80	19.71 ± 0.44 d	4.51 ± 0.11 fg	56.33 ± 1.37 d	6.50 ± 0.26 c	9.99 ± 0.63 bc	1.46 ± 0.06 cd	0.24 ± 0.01 e	81.23 ± 3.29 bcd	18.25 ± 0.80 b
	Lc-NMC	80	20.52 ± 0.55 d	4.43 ± 0.14 fg	55.33 ± 1.77 d	3.73 ± 0.20 cd	6.97 ± 0.50 bcd	0.86 ± 0.05 de	0.17 ± 0.01 e	46.65 ± 2.46 ef	10.76 ± 0.64 bcde
Berry	Vv-SB	88	5.60 ± 0.20 e	0.94 ± 0.03 i	10.64 ± 0.38 e	0.42 ± 0.06 d	4.28 ± 0.22 cd	0.44 ± 0.06 e	0.44 ± 0.06 de	4.78 ± 0.69 g	5.04 ± 0.68 e
	Vv-SM	101	5.94 ± 0.56 e	1.21 ± 0.03 i	12.00 ± 0.33 e	0.71 ± 0.06 d	4.20 ± 0.18 d	0.51 ± 0.06 e	0.51 ± 0.06 de	6.99 ± 0.63 fg	5.75 ± 0.49 e
Drupe	Zm-DMS	90	76.87 ± 2.38 b	9.74 ± 0.34 b	108.26 ± 3.75 c	8.03 ± 1.01 bc	8.82 ± 0.56 bcd	0.83 ± 0.10 de	0.98 ± 0.14 bcd	89.19 ± 11.23 bcd	9.20 ± 1.14 cde


*Different letters indicate significant difference at *P* < 0.05, Scheffe multiple range tests.*

**FIGURE 1:**
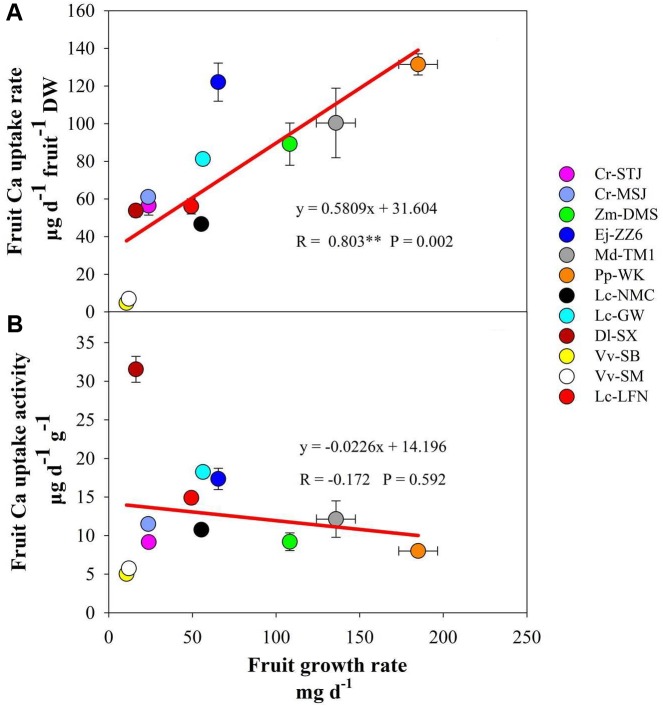
Correlation of fruit growth rate with calcium uptake rate **(A)** and calcium uptake activity **(B)** across fruit species/varieties.

Ca content per fruit is determined by fruit Ca concentration and fruit size. It was the highest in Cr-STJ and Cr-MSJ, Ej-ZZ6, and Pp-WK, but lowest in the two grapes. Fruit Ca uptake rate showed a slightly different pattern, where pomes Ej-ZZ6, Md-TM1, and Pp-WK were among the highest, followed in descending order by Zm-DMS drupe, arillate fruits (litchis and DL-SX) and citrus, while the two grape berries were the lowest Ca uptake rate. Across different fruit species/varieties, fruit growth rate and Ca uptake rate displayed a significant positive correlation (**Figure 1A**).

Fruit Ca uptake activity displayed a quiet different picture from fruit Ca uptake rate (**Table 3**). Dl-SX fruit had the highest Ca uptake activity, followed by litchis (Lc-GW, Lc-LFN, and Lc-NMC), Ej-ZZ6, Md-TM1, the citrus varieties and Zm-DMS. Grape berries had the lowest Ca uptake activity. It seemed that arillate fruits (litchis and longan) had a relatively high Ca uptake activity. Within the species of litchi, Lc-GW fruit had the highest Ca uptake activity, while Lc-NMC the lowest. Fruit Ca uptake activity had no significant correlation with fruit growth rate (**Figure 1B**).

### Ca in the Pedicel and Its Relation to Fruit Ca

**FIGURE 2:**
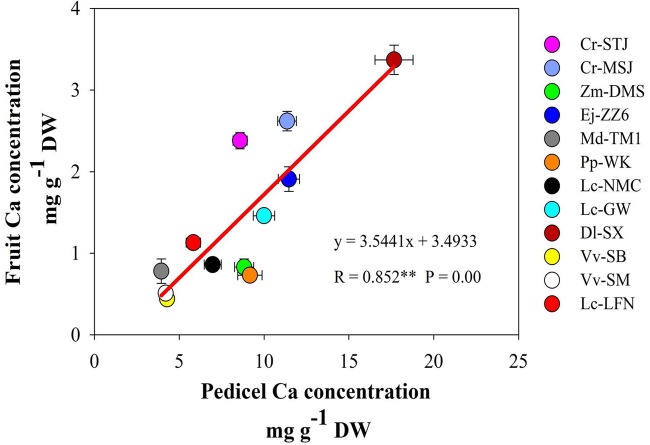
The correlation between calcium concentrations in the pedicel and in the fruit across fruit species/varieties.

It’s worth noting that Ca concentrations in the pedicel were 3–10 folds higher than those in the fruit in all the tested species/varieties. The results suggested that during the process of Ca transport toward the fruit some of the Ca was sequestered in the pedicel. The correlation between Ca concentrations in the fruit and in the pedicel differed among fruit varieties (**Table 3**). A significant positive correlation between Ca concentrations in the two parts was found in Dl-SX, Lc-GW, Lc-NMC, and Ej-ZZ6 (**Table 4**). The correlation was weak and non-significant in the other species. However, across the species/varieties, Ca concentrations in the pedicel and in the fruit had a strong and significant positive correlation (**Figure 2**), suggesting a generally synchronous accumulation of Ca in the fruit and in the pedicel.

**Table 4:** The correlations between calcium concentrations in the pedicel and the fruit in different species/varieties.

Cultivar	Date	Correlation equation	Pearson coefficient	*P*-value
Ej-ZZ6	DAFB110	*Y* = 0.2395x - 0.6653	0.823**	0.003
Md-TM1	DAFB65	*Y* = -0.0409x + 0.9375	-0.092	0.800
Pp-WK	DAFB91	*Y* = -0.0018x + 0.7459	-0.031	0.933
Cr-STJ	DAFB260	*Y* = 0.0761x + 1.7256	0.309	0.385
Cr-MSJ	DAFB228	*Y* = 0.0923x + 1.5737	0.421*	0.041
Dl-SX	DAFB107	*Y* = 0.0589x + 2.334	0.365*	0.047
Lc-LFN	DAFB76	*Y* = 0.0604x + 0.7791	0.410	0.072
Lc-GW	DAFB80	*Y* = 0.062x + 0.8405	0.614**	0.000
Lc-NMC	DAFB80	*Y* = 0.0696x + 0.3762	0.671**	0.000
Vv-SB	DAFB88	*Y* = 0.152x - 0.2067	0.561	0.091
Vv-SM	DAFB101	*Y* = 0.0161x + 0.4061	0.062	0.874


*^∗^ and ^∗∗^ indicate significant correlation at *P* < 0.05 and *P* < 0.01, respectively.*

### Pedicel Morphology and Ca Distribution

**FIGURE 3:**
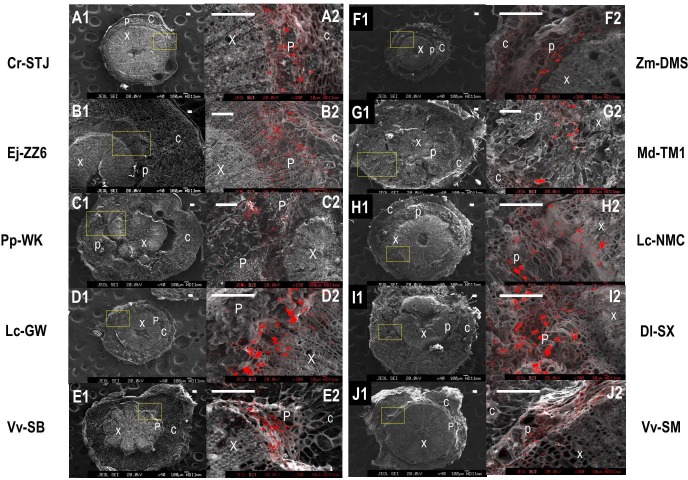
The panorama secondary electron images of the pedicels and calcium distribution image in the magnified regions of the pedicels in different fruit species/varieties. **(A1–J1)** Are the panorama pedicel images of Cr-STJ, Ej-ZZ6, Pp-WK, Lc-GW, Vv-SB, Zm-DMS, Md-TM1, Lc-NMC, Dl-SX, and Vv-SM, respectively. **(A2–J2)** Are the calcium distribution images of the magnified areas corresponding to the squared part in the panorama images of the corresponding species/varieties. “x” stands for xylem, “p” for phloem and “c” for cortex. Bars = 100 μm.

**FIGURE 4:**
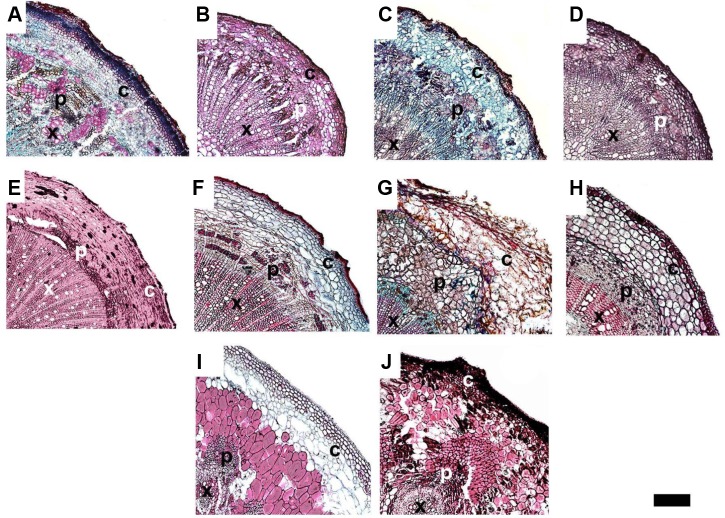
The anatomy of the pedicels in ripe fruit of different varieties. **(A–J)** Represent Dl-SX, Lc-GW, Lc-NMC, Lc-LFN, Cr-STJ, Cr-MSJ, Vv-SB, Zm-DMS, Md-TM and Pp-WK, respectively. “x” stands for xylem, “p” for phloem and “c” for cortex. Bar = 200 μm.

We used a scanning electron microscope equipped with an X-ray microanalyzer to visualize Ca distribution in the pedicel. Merging the secondary electron image and Ca mapping image shows *in situ* Ca distribution in various pedicel tissues (**Figure 3**). Anatomical characters of the pedicel are also shown in **Figure 4**. The pedicel of Zm-DMS had the smallest diameter while that of the Ej-ZZ6 was the largest among all the examined fruit species/varieties (**Figure 3**). The pedicel consisted of the pith at the very center, the vascular bundle chiefly composed of the xylem and phloem, the cortex, and the outermost epidermis (Zm-DMS and grapes) or periderm (other fruits examined) (**Figure 4**). The vascular bundles in the pedicel of most fruit species were ring-shaped, but those of the Md-TM1 and Pp-WK were club-shaped and scattered. Since, xylem is generally considered as the chief pathway for Ca transport, we focused on xylem characters. Xylem coverage, vessel number, vessel size and total vessel area in the cross sections of the pedicels are listed in **Table 5**. Both SEM and optical microscopic observations showed that apple (Md-TM1) had a poorly developed xylem with the lowest xylem coverage (12.46%). The pedicel of the two citrus cultivars, Cr-STJ and Cr-MSJ had the highest xylem coverage of over 40%. The 3 cultivars of litchis, Lc-NMC, Lc-LFN and Lc-GW, had similar xylem coverage in the range of 32.74–36.54%, while its close relative, longan (Dl-SX) had much smaller xylem coverage (19.00%). Similar to Md-TM1, Pp-WK had scattered vascular bundles, but it had higher xylem coverage (17.3%). Zm-DMS and Vv-SB had similar xylem coverage around 25%. Vessel size and number differed among the fruit varieties. The 3 litchis, Cr-MSJ and Zm-DMS had the largest vessel size, while Dl-SX, Cr-STJ and Vv-SB had the smallest vessel size. The highest vessel number was found in Lc-NMC, followed by Dl-SX and the two citrus cultivars. Md-TM1 and Zm-DMS had the lowest vessel number. The total vessel area in cross section of pedicel was the highest in Cr-MSJ and Lc-NMC, followed by the other two cultivars of litchi, while the other fruits varied around 0.02–0.03 mm^2^.

**Table 5:** Characters of the pedicel anatomy and xylem tissues in different fruit varieties.

Cultivar	Days after full bloom (DAFB)	Pedicel cross area (mm^2^)	Xylem area (mm^2^)	Xylem coverage (%)	Number of vessels	Vessel density (no./mm^2^)	Single vessel area (μm^2^)	Total vesselarea (mm^2^)
Dl-SX	AFB107	5.80 ± 1.08 a	1.15 ± 0.25 b	19.73 ± 1.50 ef	244.67 ± 67.85 bcd	48.65 ± 19.24 b	100.84 ± 6.87 c	0.03 ± 0.01 c
Lc-GW	AFB80	2.08 ± 0.22 cd	0.76 ± 0.10 bc	36.16 ± 1.33 abc	178.67 ± 21.46 cde	89.49 ± 18.59 ab	336.69 ± 46.49 a	0.06 ± 0.01 b
Lc-NMC	AFB80	3.83 ± 0.90 b	1.32 ± 0.37 b	33.71 ± 1.89 bcd	521.33 ± 27.06 a	154.48 ± 42.49 a	175.85 ± 10.65 c	0.09 ± 0.01 a
Lc-LFN	AFB77	2.46 ± 0.68 bcd	1.33 ± 0.52 b	48.18 ± 10.94 a	179.33 ± 3.18 cde	91.71 ± 33.64 ab	303.08 ± 23.06 ab	0.05 ± 0.00 b
Cr-STJ	AFB249	1.63 ± 0.38 d	0.78 ± 0.21 bc	47.32 ± 1.59 a	257.33 ± 38.46 bc	177.67 ± 52.65 a	151.37 ± 70.86 c	0.04 ± 0.02 bc
Cr-MSJ	AFB249	6.72 ± 0.08 a	2.77 ± 0.03 a	41.17 ± 0.31 ab	274.67 ± 10.41 b	40.90 ± 2.01 b	384.52 ± 10.26 a	0.11 ± 0.00 a
Vv-SB	AFB88	3.76 ± 0.32 bc	0.88 ± 0.04 bc	23.58 ± 0.94 def	157.33 ± 23.70 de	42.44 ± 7.77 b	167.68 ± 27.89 c	0.03 ± 0.00 c
Zm-DMS	AFB90	1.04 ± 0.23 d	0.26 ± 0.07 c	24.89 ± 0.92 cde	88.00 ± 16.65 e	100.57 ± 36.69 ab	209.42 ± 43.27 bc	0.02 ± 0.00 c
Md-TM	AFB53	7.30 ± 0.47 a	0.91 ± 0.31 bc	12.09 ± 3.88 f	134.00 ± 13.53 e	18.51 ± 2.16 b	184.39 ± 50.21 c	0.03 ± 0.01 c
Pp-WK	AFB65	6.70 ± 0.02 a	1.16 ± 0.19 b	17.35 ± 2.81 ef	170.00 ± 13.01 cde	25.36 ± 1.87 b	141.43 ± 26.59 c	0.02 ± 0.01 c


*Different letters indicate significant difference at *P* < 0.05, Scheffe multiple range tests.*

Ca mapping with electron probe showed that Ca was not evenly distributed across the pedicel. In all the tested fruit species/varieties, Ca was more distributed in the phloem tissue than in the xylem tissue. Ca was most abundant in the phloem cells adjacent to the xylem (cambium). There were a lot of “Ca particles” scattered in the phloem, possibly corresponding to cells containing Ca oxalate. The results suggested flux of Ca to the phloem from the xylem during the process of Ca transport in the pedicel. Apparently, Dl-SX and Lc-GW had denser Ca particles in the phloem than the other fruit species/varieties examined.

### Xylem Functionality Observation

**FIGURE 5:**
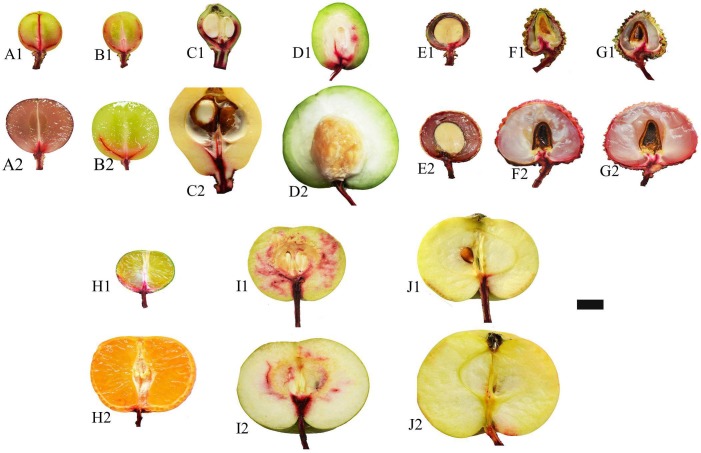
Xylem functionality observations with dye tracing in different fruit species/varieties at early maturing stage and ripe stage. **(A–J)** Represent Vv-SB, Vv-SM, Zj-ZZ6, Zm-DMS, Dl-SX, Lc-NMC, Lc-GW, Cr-STJ, Pp-WK, and Md-TM1, respectively, at early maturing stage when followed by 1 and at ripe stage by 2. Bar = 1 cm.

The continuity or functionality the xylem vessels was analyzed by using 1% w/v safranin O dye tracing. The results (**Figure 5**) showed that dye solution fed through fruit stalk had a greater coverage in the fruit tissues at the early maturing stage than at the ripe stage in all species/varieties examined. In ripe fruits of most species, the dye transport to the fruit was halted at the joining site of the fruit and the pedicel, indicating the site of vessel function loss. The results suggest that it is a general phenomenon that fruit xylem vessel gradually lose function with fruit maturation. Among the fruits examined, the pome of apple (Md-TM1) had the poorest vessel functionality, as no dye fed from the stalk could be observed in the fruit tissues even at the early maturing stage. The poor distribution of dye agreed with the poor development of xylem shown in **Figures 3**, **4**. The result also suggested that fruit vessel function loss occurred earlier in Md-TM1 than the other species/varieties and that apple fruit might be a phloem-fed fruit during maturation. Two other pomes, Pp-WK and Ej-ZZ6, had a much better vessel functionality than apple, and the dye fed through the fruit stalk was relatively evenly distributed in their flesh tissue at the early maturing stage. In citrus and the arillate fruits of litchi and longan, dye could not enter the flesh tissue, where there was no vascular bundle, but was extensively distributed in the skin tissue with abundant vascular bundles. In the stone fruit of Zm-DMS, the dye in the early maturing fruit was found in tissues around the stone, with less distribution in the outer flesh parts, while in ripe fruit, no dye was transported into the fruit, indicating a complete “cut-off” of the vessel system at the fruit base.

### Relations of Pedicel Xylem Parameters to Fruit Ca Uptake Capacity

Across the fruit species/varieties, neither fruit Ca uptake rate nor fruit Ca uptake activity had a significant correlation with xylem area, xylem coverage, vessel number, vessel size or the total vessel area in the cross section of the pedicel (**Table 6**). The results suggest that the xylem morphology in the pedicel does not seem to create a rate limiting effect on fruit Ca uptake.

**Table 6:** Cross-species correlations of xylem parameters with fruit calcium uptake capacity.

Fruit calcium uptake capacity (y)	Xylem parameter (x)	Linear regression equation	Correlation coefficient	*P*-value
Calcium uptake rate (μg d^-1^)	Xylem area (μm^2^)	*Y* = -6.887x + 75.919	-0.131	0.717
	Xylem coverage (%)	*Y* = -0.9816x + 97.975	-0.363	0.303
	Vessels number	*Y* = -0.0955x + 89.177	-0.334	0.346
	Vessel density (no./mm^2^)	*Y* = -0.1766x + 82.064	-0.278	0.437
	Single vessel area (μm^2^)	*Y* = -0.0027x + 68.71	-0.007	0.984
	Total vessel area (μm^2^)	*Y* = -263.49x + 80.42	-0.281	0.431
Calcium uptake activity (μg d^-1^ g^-1^)	Xylem area (μm^2^)	*Y* = 0.6659x + 12.293	0.059	0.871
	Xylem coverage (%)	*Y* = -0.0457x + 14.438	-0.078	0.830
	Vessels number	*Y* = 0.0045x + 12.054	0.073	0.842
	Vessel density (no./mm^2^)	*Y* = -0.0169x + 14.381	-0.123	0.735
	Single vessel area (μm^2^)	*Y* = -0.0056x + 14.262	-0.070	0.847
	Total vessel area (μm^2^)	*Y* = -6.95x + 13.372	-0.009	0.980


## Discussions

### Difference in Ca Uptake Capacity Among Fruits

In the present study, we examined fruit Ca uptake in 12 species/varieties. However, in order to collected fruits of different species, we needed to take samples from different orchards across regions with different soil and climate conditions and culture practices, which exert significant influence on fruit Ca uptake (Saure, 2005; Montanaro et al., 2014) and thus complicate the cross-species comparison. Despite that, we can still see some general patterns in fruit Ca uptake in different species/varieties from our results. Fruit species with larger fruit size (e.g., pomes) tended to have a higher Ca uptake rate than those with smaller fruit size (e.g., grape berries), and there was a strong positive correlation between fruit Ca uptake and fruit growth rates (**Figure 1A**). Tromp (1979) showed a positive correlation between fruit growth and Ca accumulation among individual fruit of apple and concluded that fruit growth was a key determinant of fruit Ca uptake. Our results support his conclusion and suggested that this might be true across fruit species/varieties. VPD exerts a strong effect on fruit Ca gain, as it drives transpiration (Montanaro et al., 2010, 2015). In this study, the average VPDs during the development periods of all the tested fruits were all below 1.0 (0.391–0.853) (**Table 1**). Montanaro et al. (2015) found that when VPD was very low, fruit Ca gain is more contributed by growth instead of transpiration. The close relation between Ca uptake and growth in fruit suggests fruit Ca uptake is largely demand-determined.

Different cultivars of the same species (e.g., litchi, citrus and grape) had Ca uptake rates within a much narrower range compared with the cross-species range. However, significant difference in Ca uptake rate was found among different litchi cultivars (e.g., Lc-NMC vs. Lc-GW) grown in the same orchard under the same soil and climate conditions and culture practices. The results suggest that fruit Ca uptake capacity has a strong genetic background, which is the “behind-curtain” determinant of the permeance of fruit skin, the conductivity of Ca transport pathways as well as fruit size and growth rate.

However, during fruit development, rapid fruit expansion growth promoted by gibberellins, has been found associated with reduced Ca accumulation due to the suppression effect of gibberellins on Ca transport (Saure, 2005). Therefore, Ca deficiency frequently develops during vigorous growth. In this contest, Ca uptake rate relative to growth is a better index to show the balance between fruit Ca supply and the demand for growth. In this study, we adopted a novel parameter, fruit Ca uptake activity, Ca uptake rate per unit fruit mass, to reflect Ca uptake relative to growth (dry mass accumulation). The small arillate fruit of longan (Dl-SX) had a significantly higher fruit Ca uptake activity than the other species examined, while grapes had the lowest despite their smallest fruit size. Across species and varieties, fruit Ca uptake activity had no significant correlation with growth rate. Among the three litchi cultivars, Lc-GW and Lc-LFN had a higher Ca uptake activity than Lc-NMC (**Table 3**). The former two are less susceptible to fruit cracking and have a better postharvest performance than Lc-NMC (Fan et al., 2014; Huang et al., 2005a,b). Therefore, Ca uptake activity may be a good index to reflect the susceptibility to disorders associated with Ca deficiency in a given species.

As for Ca nutrition value of table fruits, flesh Ca concentration is more important than Ca content in the whole fruit, which contains non-edible tissues such as stones and leathery skin. Our study showed that loquat and citrus had the highest Ca content in the flesh and can be a good source for Ca nutrition. The Ca concentration in the flesh or aril of the litchis was the lowest. The results suggest that partitioning of imported Ca among fruit parts differs among fruit types.

### Xylem Functionality and Pathways of Ca Transport to Fruit

Fruit is a terminal organ with almost all nutrients delivered from the tree through the connecting pedicel via xylem/apoplast pathway and/or phloem/ symplast pathway or both pathways. It is generally accepted that Ca enters fruit exclusively through the xylem/apoplast pathway as Ca in phloem/symplast is maintained at a very low level (Saure, 2005; Tonetto de Freitas and Mitcham, 2012; Hocking et al., 2016). Therefore fruit Ca uptake is believed to be determined by Ca concentration in the xylem sap as well as xylem sap influx, which is related to fruit transpiration and growth (Montanaro et al., 2014; Hocking et al., 2016). Factors influencing xylem sap flow into the fruit, such as fruit transpiration (Montanaro et al., 2010, 2014) and xylem functionality (Dichio et al., 2003; Miqueloto et al., 2014), impact fruit Ca uptake. In many fruits, such as kiwi (Dichio et al., 2003), apple (Miqueloto et al., 2014) and grape berry (Düring et al., 1987; Coombe and McCarthy, 2000; Choat et al., 2009), vessel functionality showed dynamic changes due to balance between xylem functionality loss caused by vessel stretching during fruit expansion and vessel functionality recovery due to formation of new vessel, and at the late stage of fruit development, permanent loss of xylem functionality occurs. Time of vessel functionality loss in apple was found to differ according to cultivars, occurring at much earlier stage in ‘Catarina’ than in ‘Fuji,’ which explains the high susceptibility to bitter pit disorder in ‘Catarina’ (Miqueloto et al., 2014). The dynamics of vessel functionality has been shown to satisfactorily explain fruit Ca accumulation pattern (Dichio et al., 2003; Mazzeo et al., 2013; Miqueloto et al., 2014). Results in this study show that xylem functionality loss with fruit ripening is a universal phenomenon and that the site of xylem functionality loss is at the fruit-pedicel joint (**Figure 5**). Apple was shown to have weakest xylem functionality (**Figure 4**). In agreement with this, apple pedicel had the least development of vascular bundle among tested fruits (**Figures 3**, **4**). However, xylem functionality difference among species/varieties could not satisfactorily explain the difference in fruit Ca capacity. For example, apple had the poorest vessel functionality and least development in the secondary xylem, but its fruit Ca uptake rate was among the highest. Jones et al. (1983) estimated the Ca accumulation in apple fruit based on vessel influx volume and Ca concentration and found great discrepancy between the estimated value and measured Ca accumulation. One of the explanations to this discrepancy was that phloem might also be a major pathway for Ca transport (Jones et al., 1983). There are studies showing that both the xylem and phloem participated in Ca movement into fruit (Himelrick and McDuffie, 1983; Yang and Jie, 2005; Song et al., 2018). Himelrick and McDuffie (1983) suggested that Ca transport into fruit was likely via the phloem but movement of Ca inside fruit was through the xylem. Our previous study also provided evidences showing important contribution of phloem pathway to fruit Ca uptake in litchi Song et al. (2018). Brauer et al. (1998) found the concentration of Ca in phloem sap in a range of 10 to 100 μM, which is far higher than that reported in cytosol. In the pedicels of all the fruits examined, phloem had a greater Ca abundance than xylem (**Figure 3**). Therefore, Ca transport to fruit via phloem/symplast pathway cannot be ruled out, especially in fruits like apple, whose xylem dysfunction occurs very early during fruit development (Miqueloto et al., 2014; **Figure 5**).

The present study shows that it is a universal phenomenon among plant species that pedicel has a higher Ca concentration than the fruit, although the pedicel-fruit Ca concentration gradient (3–10 times) differs among genotypes. In previous studies in litchi, we found that Ca concentration in the pedicel was ten times higher than in the fruit pericarp, and suggested that there might be a “bottleneck effect” of Ca transport in the pedicel (Huang et al., 2006; Song et al., 2018), which results in more Ca accumulation in the pedicel when more Ca is transported toward the fruit. Thus a positive correlation between Ca in the pedicel and in the fruit should be found. In this study, across species/varieties as well as within Dl-SX, Ej-ZZ6, Lc-GW, and Lc-NMC, Ca concentrations in the pedicel and the fruit displayed a good positive correlation (**Figure 1** and **Table 4**). The results suggest a generally synchronous accumulation of Ca in the fruit and in the pedicel. However, in the other species/varieties beyond Dl-SX, Ej-ZZ6, Lc-GW, and Lc-NMC, the correlation between calcium concentrations in the pedicel and the fruit was not significant. It is likely that the Ca accumulated in the pedicel might be remobilized to the fruit via phloem pathway, which breaks the correlation. Further studies are needed to understand the change patterns of the quantity and forms of Ca in the phloem of pedicel and to clarify its relation with fruit Ca uptake.

### Relations of Fruit Ca Uptake Capacity With Xylem Characters in the Pedicel

Xylem sap influx, one of the key determinants of fruit Ca uptake capacity (Saure, 2005; Hocking et al., 2016), is determined by xylem hydraulic conductivity, which itself is a function of vessel size (Tyree and Ewers, 1991; Zach et al., 2010). In this study, neither fruit Ca uptake rate nor fruit Ca uptake activity was found to have a significant correlation with pedicel xylem anatomical characters, including xylem area, xylem coverage, vessel density, total vessel area and vessel size in the pedicel across the tested fruit species and varieties. The results indicate that these xylem characters in the pedicel are not a rate-limiting factor in Ca delivery to the fruit. Dye tracing showed that in most fruits xylem functionality loss occurred at the fruit-pedicel joining site as fruit matured, indicating this site might be a rate-limiting “bottleneck” in Ca transport to fruit. Indeed, Mazzeo et al. (2013) found that hydraulic resistance in the receptacle portion, that included the fruit-pedicel joint, was the highest in kiwi. Further anatomical study in relation to fruit Ca uptake capacity should focus on this site.

## Conclusion

The following conclusions could be drawn based on our results and the above discussions. (1) fruit growth may be a dominant determinant of Ca uptake; (2) the universal pattern of Ca being higher in the pedicel than in the fruit indicates existence of a pedicel-fruit “bottleneck” effect in Ca transport across species; (3) xylem functionality loss with fruit maturation is also a universal event; (4) in the pedicel, Ca is generally more distributed in the phloem; (5) vessel morphology in the pedicel is not rate-limiting for fruit Ca uptake; (6) phloem pathway might contribute to fruit Ca uptake.

## Author Contributions

WS, JY, and OK performed the field experiments and lab analyses, data processing, and contributed draft writing. HW and XH contributed to the experimental design, research fund, and critical revising of the manuscript.

## Conflict of Interest Statement

The authors declare that the research was conducted in the absence of any commercial or financial relationships that could be construed as a potential conflict of interest.

## Supplementary Material

FIGURE S1 Changes in VPD in different sampling sites. **(A)** Hourly VPD change in Zhajiang, where loquat fruit were collected; **(B)** Hourly VPD change in Guizhou, where apple and pear fruits were collected; **(C)** Daily VPD change in Shenzhen, where litchi and longan fruit were sampled. Bars indicate fruit development periods.

FIGURE S2 Hourly changes in VPD in South China Agricultural University, Guangzhou. Bars indicate development periods of fruits sampled.
