# Supplementary figures and images for: Linking Fruit Ca Uptake Capacity to Fruit Growth and Pedicel Anatomy, a Cross-Species Study
Source: Front Plant Sci. 2018 May 9;9:575. doi: 10.3389/fpls.2018.00575 (PMC5954447; doi:10.3389/fpls.2018.00575)

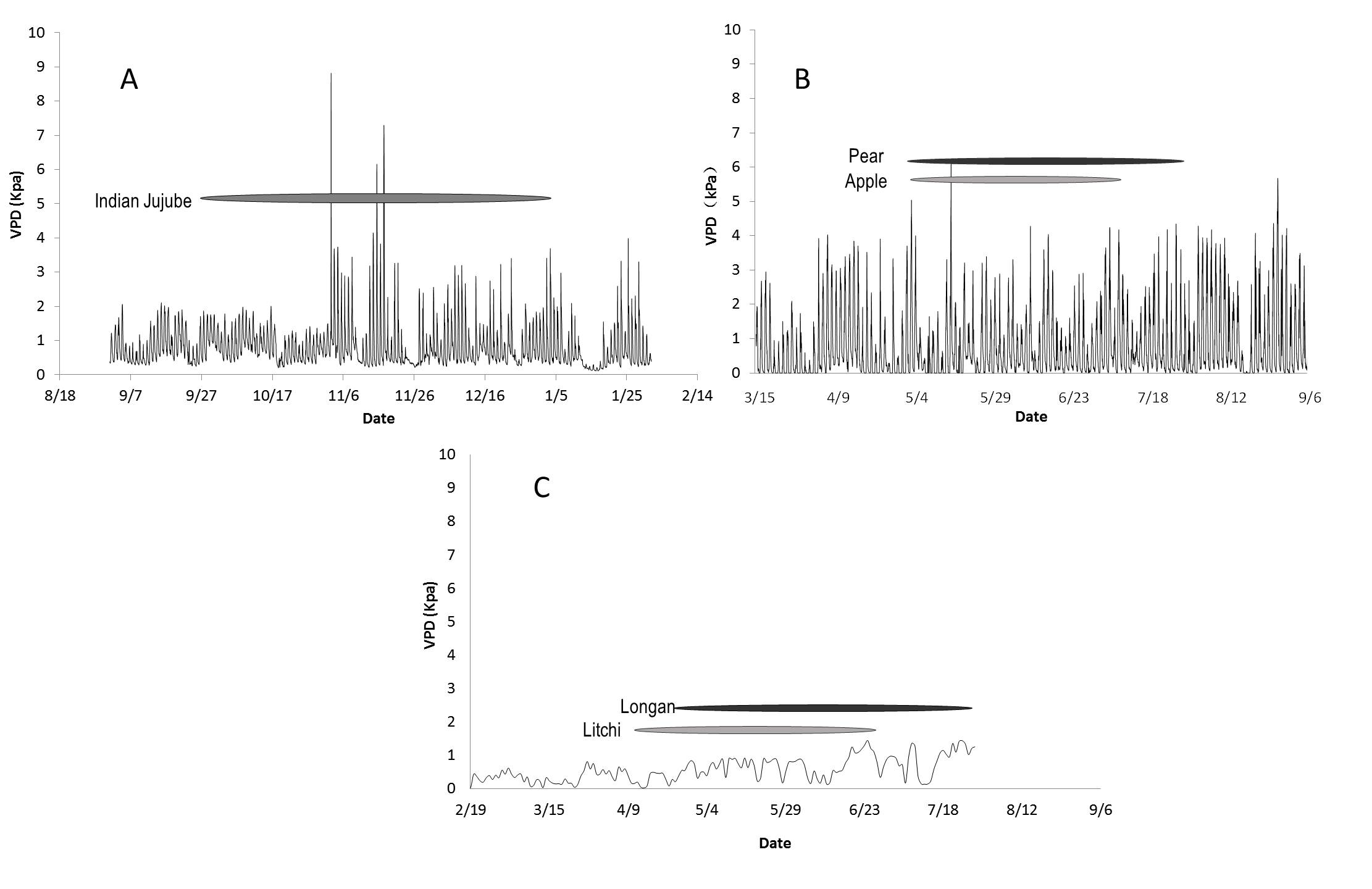

Supplement: FIGURE S1 — Changes in VPD in different sampling sites. (A) Hourly VPD change in Zhajiang, where loquat fruit were collected; (B) Hourly VPD change in Guizhou, where apple and pear fruits were collected; (C) Daily VPD change in Shenzhen, where litchi and longan fruit were sampled. Bars indicate fruit development periods. [file Image_1.JPEG]

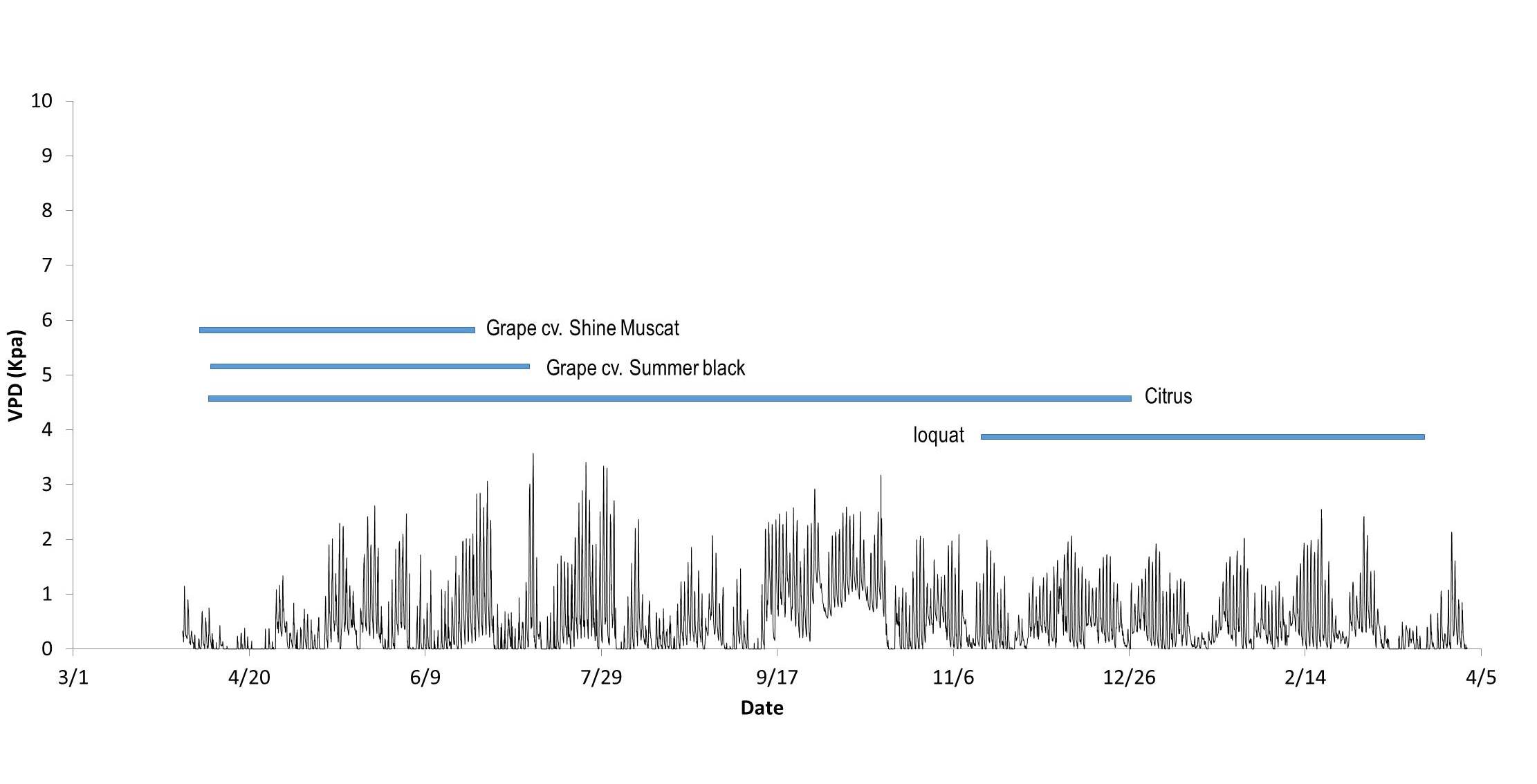

Supplement: FIGURE S2 — Hourly changes in VPD in South China Agricultural University, Guangzhou. Bars indicate development periods of fruits sampled. [file Image_2.JPEG]
